# Zinc and vitamin C intake increases spike and neutralising antibody production following SARS‐CoV‐2 infection

**DOI:** 10.1002/ctm2.731

**Published:** 2022-02-20

**Authors:** Amy May Lin Quek, Delicia Shu Qin Ooi, Ooiean Teng, Chang Yien Chan, Geelyn Jeng Lin Ng, Mei Yen Ng, Sidney Yee, Ee Wan Cheong, Ruifen Weng, Alex R. Cook, Mikael Hartman, Veronique Angeli, Paul Anantharajah Tambyah, Raymond Chee Seong Seet

**Affiliations:** ^1^ Department of Medicine Yong Loo Lin School of Medicine National University of Singapore Singapore; ^2^ Division of Neurology Department of Medicine National University Hospital Singapore; ^3^ Department of Pediatrics Yong Loo Lin School of Medicine National University of Singapore Singapore; ^4^ Khoo Teck Puat‐National University Children's Medical Institute National University Hospital National University Health System Singapore; ^5^ Diagnostic Development Hub, Agency for Science Technology and Research (A*STAR) Singapore; ^6^ Saw Swee Hock School of Public Health National University of Singapore and National University Health System Singapore; ^7^ Department of Surgery Yong Loo Lin School of Medicine National University of Singapore Singapore; ^8^ Immunology Translational Research Programme Department of Microbiology and Immunology Yong Loo Lin School of Medicine National University of Singapore Singapore; ^9^ Immunology Programme Life Sciences Institute National University of Singapore Singapore; ^10^ Division of Infectious Diseases National University Hospital Singapore; ^11^ Infectious Diseases Translational Research Program, Yong Loo Lin School of Medicine National University of Singapore Singapore; ^12^ Healthy Longevity Translational Research Program Yong Loo Lin School of Medicine National University of Singapore Singapore

Dear Editor,

Previous studies have not examined whether pharmacologic interventions could increase SARS‐CoV‐2 antibody responses. Certain medications (e.g. zinc and vitamin C) are known to stimulate immunologic responses following infections.^1^, ^2^ Zinc exerts pluripotent effects on the immune system and supports the integrity of the epithelial cell barriers,^1^, ^3^ while vitamin C is an antioxidant that potentially protects against viral respiratory infections.^2^ Hydroxychloroquine and ivermectin are anti‐parasitic medications that are known to modulate innate and adaptive immunity.^4^, ^5^ By contrast, povidone‐iodine is a topical broad spectrum antiseptic capable of direct virucidal effects.^6^ We hypothesise that interventions that support immune regulatory functions could enhance production of anti‐SARS‐CoV‐2 spike and neutralising antibodies among individuals with prior infection. Using materials and resources of the DORM trial (NCT04446104),^7^ we compared the antibody responses at baseline and on day 42 among seropositive participants who received the different medications as part of this trial.

Participants from the study were selected from the DORM trial, an open label, randomised clinical trial that examined the efficacy of either oral hydroxychloroquine (400 mg followed by 200 mg/day), povidone‐iodine throat spray (three times a day, approximately 270 μg/day), oral ivermectin (12 mg, single dose), oral zinc + vitamin C (80 mg zinc sulfate, 500 mg vitamin C/day) or oral vitamin C (500 mg/day), for 42 days to reduce SARS‐CoV‐2 infection (Supporting Information).^7^ From 4257 recruited participants, those found with new SARS‐CoV‐2 infection on recruitment were enrolled into the present substudy.

Antibody titers were measured using two different assays that quantified binding (Elecsys®, Roche, Germany) and neutralising antibodies (cPass™, GenScript, USA) targeting the SARS‐CoV‐2 spike antigens. To examine the impact of different interventions on the immune cells, the frequency of B and T lymphocytes was analysed using pre‐formulated DURAClone IM Phenotyping Basic and B Cell Panels (Beckman, USA). Serum zinc was measured in zinc‐treated participants using an inductively coupled plasma mass spectrometry method (PerkinElmer, USA). SPSS Statistics version 27 (IBM Corporation, Armonk, USA) was used for all analysis (Supporting Information).

A total of 422 men were enrolled, from among 478 seropositive cases in the DORM trial; those excluded either withdrew from the study (*n* = 22) or did not return for follow‐up visit (*n* = 24) (Figure S1). The primary cohort comprised 422 men (mean age, 33.0 years; standard deviation (SD), 7.3 years) who received zinc + vitamin C (*n* = 68), hydroxychloroquine (*n* = 67), ivermectin (*n* = 99), povidone‐iodine (*n* = 107) and vitamin C (*n* = 81) (Table 1, Figure S1). Few had medical co‐morbidities (hypertension, 1.4%; diabetes mellitus, 0.7%; hyperlipidemia, 0.2%). Overall, anti‐SARS‐CoV‐2 spike antibody positivity increased from baseline level of 80% to 94% by day 42, whereas neutralising antibody positivity increased from 44% to 49% by day 42.

**TABLE 1:** Clinical characteristics of study participants

	Zinc + vitamin C (*n* = 68)	Hydroxychloroquine (*n* = 67)	Ivermectin (*n* = 99)	Povidone‐iodine (*n* = 107)	Vitamin C (*n* = 81)
Participant characteristics					
Age (years), mean (SD)	33.2 (7.8)	30.6 (6.4)	33.6 (6.9)	32.0 (6.6)	32.9 (7.1)
Country of origin					
Bangladesh	36 (52.9%)	28 (41.8%)	46 (46.5%)	52 (48.6%)	40 (49.4%)
India	32 (47.1%)	38 (56.7%)	51 (51.5%)	55 (51.4%)	41 (50.6%)
Others	0	1 (1.5%)	2 (2.0%)	0	0
Medical history					
Hypertension	1 (1.5%)	1 (1.5%)	3 (3.1%)	1 (0.9%)	0
Diabetes mellitus	1 (1.5%)	0	1 (1.0%)	0	1 (1.3%)
Hyperlipidemia	0	0	1 (1.0%)	0	0
Baseline parameters					
Systolic BP (mmHg)	136.6 (15.9)	127.4 (11.6)	135.7 (15.1)	134.4 (17.7)	133.8 (17.5)
Diastolic BP (mmHg)	86.6 (11.6)	81.2 (6.4)	89.8 (9.3)	86.6 (11.6)	88.5 (10.9)
Pulse rate (per min)^a^	97.4 (15.5)	86.9 (7.9)	96.9 (13.4)	93.3 (11.8)	97.1 (13.9)
Body mass index (kg/m^2^)	24.34 (3.43)	24.36 (3.44)	25.73 (2.72)	24.62 (3.55)	24.19 (3.02)

^a^Resting heart rate.

Anti‐SARS‐CoV‐2 immunoglobulin G (IgG) levels, which were comparable at baseline, increased substantially by day 42, especially in the zinc + vitamin C group compared with other interventions (Figure 1A,B). By contrast, an increase in neutralising antibodies (measured by percent inhibition of surrogate virus neutralisation tests) was confined to the zinc + vitamin C group by day 42 (Figure 1C,D). Among seropositive men without neutralising antibodies at study entry, conversion to neutralising antibody positivity was highest in the zinc + vitamin C group (46.7%) by day 42, compared with vitamin C (19.6%), hydroxychloroquine (30.8%), ivermectin (20%) and povidone‐iodine (9.8%) (*p* < .001, chi‐squared test) (Figure 2). Overall, seropositive men who were initially negative for neutralising antibodies were approximately four times more likely to develop neutralising antibody positivity by day 42 in the zinc + vitamin C group compared with other interventions (odds ratio (OR) 3.75, 95% confidence interval (CI) 1.69–8.32). To further investigate the primary findings, zinc was measured in baseline and day 42 sera of men who received zinc + vitamin C. As expected, serum zinc rose from a baseline mean of 14.0 mmol/l (SD 1.9) to 17.8 mmol/l (SD 2.5) by day 42. The extent of rise in serum zinc from baseline to day 42 correlated significantly with anti‐SARS‐CoV‐2 spike IgG (*r* = 0.401, *p* = .001) (Figure 3A), but not with neutralising antibodies (Figure 3B).

**FIGURE 1:**
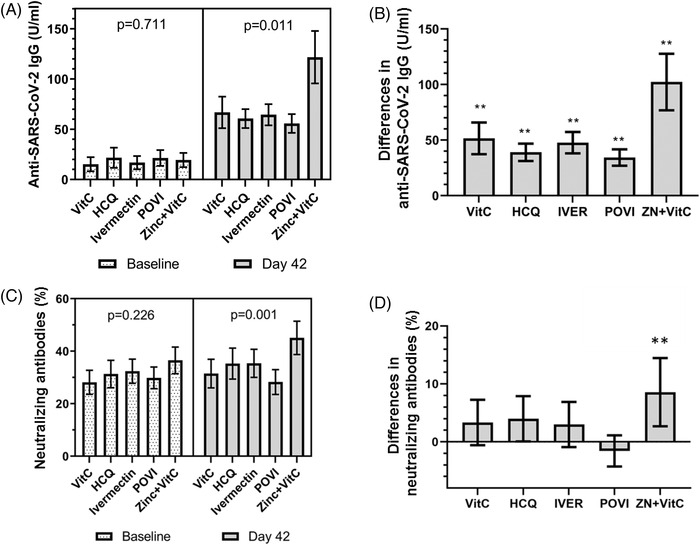
Comparison in SARS‐CoV‐2‐specific antibodies between intervention groups. (A) Despite comparable baseline levels of anti‐SARS‐CoV‐2 spike immunoglobulin G (IgG) across different interventions, spike IgG increased considerably by day 42 especially in the zinc + vitamin C group. Adjusted *p*‐value for day 42 comparisons was .042 (accounting for age, country of origin and medical history). (B) Differences in anti‐SARS‐CoV‐2 spike IgG were highest in the zinc + vitamin C group compared with other interventions. (C and D) Despite comparable neutralising antibodies at baseline, a significant increase in neutralising antibodies was observed in the zinc + vitamin C group in contrast with other interventions. Adjusted *p*‐value for day 42 comparisons was .020 (accounting for age, country of origin and medical history). *p*‐Values presented in the figure were unadjusted. Neutralising antibodies were measured as percent inhibition using a surrogate virus neutralisation test (%). Bar graphs (error bars) denote median values with 95% confidence intervals. Asterisks (**) denote *p* < .0125 when a one‐sample Kolmogorov–Smirnov test was performed against a test value of 0. Abbreviations: VitC, vitamin C; HCQ, hydroxychloroquine; POVI, povidone‐iodine; IVER, ivermectin; ZN, zinc

**FIGURE 2:**
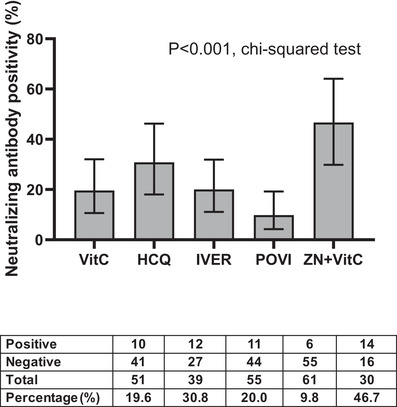
Comparison in proportion of participants who developed neutralising antibodies by day 42 according to intervention. Bar graphs (error bars) denote median values with 95% confidence intervals. The 95% confidence intervals of proportion were estimated using a one‐sample proportion test with a hypothesised value of 0.5. *p*‐Value was derived chi‐squared test. The table below the figure indicate the number of participants with positive and negative neutralising antibodies, number of participants in each intervention without neutralising antibodies at baseline, and percentage conversion in neutralising antibodies (%). Abbreviations: VitC, vitamin C; HCQ, hydroxychloroquine; POVI, povidone‐iodine; IVER, ivermectin; ZN, zinc

**FIGURE 3:**
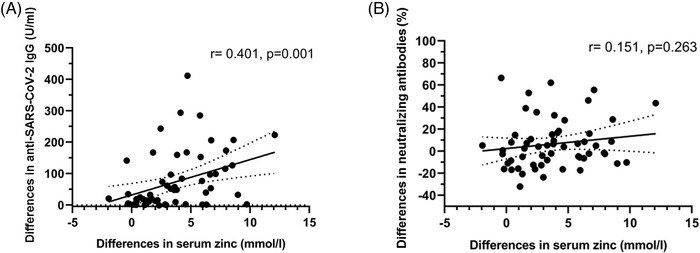
Scatter plot comparing the association between differences in serum zinc and (A) anti‐SARS‐CoV‐2 spike immunoglobulin G (IgG) and (B) neutralising antibodies, at baseline and day 42. Neutralising antibodies were measured as percent inhibition using a surrogate virus neutralisation test (%). Solid line on each figure indicates a linear regression line and its 95% confidence interval (dotted line). Spearman correlation methods were used to derive correlation factor (*r*) and *p*‐values

To examine the impact of the different interventions on immune cell populations, immunophenotyping was performed on whole blood in 211 participants (Figure S2). Compared with other interventions, zinc + vitamin C‐treated men had a higher percentage of transitional B cells (mean 3.49% vs. 2.54%, *p* < .001) (Table S1). No statistically significant differences were observed with isotype class switch, marginal zone, naïve and plasmablast B cells, CD4+ T cells, CD8+ T cells, natural killer cells, classical, intermediate and non‐classical monocytes. In this cohort, transitional B cells were weakly correlated with neutralising antibodies (*r* = 0.192, *p* = .005) (Figure S3A). Among men who were initially negative for neutralising antibodies, an increase in double‐negative T cells frequency was observed among those with seroconversion in neutralising antibodies by day 42 (mean 8.40% vs. 5.92%, *p* = .004) (Table S2). A weak but statistically significant association was observed between double‐negative T cells and neutralising antibodies by day 42 (*r* = 0.272, *p* = .004) (Figure S3B).

Findings from this study highlight suboptimal production of neutralising antibodies in more than 50% of individuals following an asymptomatic infection. Compared with other interventions, those who received zinc + vitamin C were found to subsequently mount a greater antibody response. Among individuals who were initially seronegative to neutralising antibodies at study entry, conversion to positivity was approximately four times higher among zinc‐treated men compared with those who received other interventions. By activating innate and adaptive B and T cellular responses, the immune system produces binding antibodies especially against the nucleocapsid and spike proteins of the SARS‐CoV‐2 virus, and neutralising antibodies to aid immunity.^8^, ^9^ We observed significant alterations in early transitional B cells but not in B cell populations at later stages of maturation. The significance of increase in double‐negative T cells among neutralising antibody seroconverters is unclear. Among hospitalised patients, those with a higher double‐negative T cells to surface expression of CD4 and CD8, tended to have a milder disease with fewer requiring oxygen and ventilatory support,^10^ suggesting double‐negative T cells could be beneficial during SARS‐CoV‐2 infection.

To our knowledge, this study is the first to demonstrate the efficacy of oral zinc + vitamin C treatment to stimulate antibody production following SARS‐CoV‐2 infection. Future studies should examine whether increase in antibody production following zinc + vitamin C could sustain immunity against re‐infection, and enhance antibody responses following vaccination among immunocompromised individuals. Results from this study should be confirmed in prospectively designed studies.

## CONFLICTS OF INTEREST

Dr. Seet reported receiving grants from the National Medical Research Council and Temasek Foundation, Singapore. Dr. Tambyah reported receiving grants from Johnson and Johnson, GlaxoSmithKline and Roche.

## Supporting information

Supporting Information

**Figure S1**. Screening and randomisation. From the 4257 men who were assessed for eligibility, 3835 were excluded: 3789 were seronegative on recruitment, 22 withdrew from study and 24 did not return for follow‐up visit. A total of 422 men formed the primary cohort (68 received oral zinc + vitamin C, 67 oral hydroxychloroquine, 99 oral ivermectin, 107 povidone‐iodine throat spray and 81 oral vitamin C)
**Figure S2**. Characterisation of B and T cell populations by flow cytometric methods to derive (A) transitional, class unswitched and switched, marginal zone, naïve B cells and plasmablasts, and (B) CD4+ helper, CD8+ cytotoxic, double‐negative, double‐positive, natural killer T cells, natural killer cells, classical, intermediate and non‐classical monocytes. Double‐negative and double‐positive T cells refer to surface expression of CD4 and CD8
**Figure S3**. (A) Scatter plot comparing the association between transitional B cells and neutralising antibodies in a subset of individuals with immunophenotyping data (*n* = 211). (B) Scatter plot comparing the association between double‐negative T cells and neutralising antibodies by day 42 among participants without neutralising antibodies at baseline. Neutralising antibodies were measured as percent inhibition using a surrogate virus neutralisation test (%). Solid line on each figure indicates a linear regression line and its 95% confidence interval (dotted line). Spearman correlation methods were used to derive correlation factor (*r*) and *p*‐values

**Table S1**. Comparison in immunophenotyping profile between zinc + vitamin C and other interventions on day 42. B and T cell populations were characterised in a proportion of participants following the 42‐day trial and summarised as percent (%)
**Table S2**. Immunophenotyping and neutralising antibodies among individuals with and without seroconversion. B and T cell populations were characterised on day 42 in men who were initially negative for neutralising antibodies and summarised as percent (%). Neutralising antibodies were measured as percent inhibition using a surrogate virus neutralisation test (%)
