# Supplementary material for: Zinc and vitamin C intake increases spike and neutralising antibody production following SARS‐CoV‐2 infection
Source: Clin Transl Med. 2022 Feb 20;12(2):e731. doi: 10.1002/ctm2.731 (PMC8858613; doi:10.1002/ctm2.731)
Supplement: Supplementary file 1 — Supporting Information [file CTM2-12-e731-s001.docx]

**Supplementary Materials**

**Zinc and vitamin C intake increases spike and neutralizing antibody production following SARS-CoV-2 infection**

Amy May Lin Quek^1,2^, Delicia Shu Qin Ooi^3,4^, Ooiean Teng^1^, Chang Yien Chan^3,4^, Geelyn Jeng Lin Ng^1^, Mei Yen Ng^1^, Sidney Yee^5^, Ee Wan Cheong^5^, Ruifen Weng^5^, Alex R Cook^6^, Mikael Hartman^6,7^, Veronique Angeli^8,9^, Paul Anantharajah Tambyah^1,10^, Raymond Chee Seong Seet^1,2,11^

^1^ Department of Medicine, Yong Loo Lin School of Medicine, National University of Singapore, Singapore

^2^ Division of Neurology, Department of Medicine, National University Hospital, Singapore

^3^ Department of Pediatrics, Yong Loo Lin School of Medicine, National University of Singapore, Singapore

^4^ Khoo Teck Puat-National University Children’s Medical Institute, National University Hospital, National University Health System, Singapore

^5^ Diagnostic Development Hub, Agency for Science, Technology and Research (A*STAR), Singapore.

^6^ Saw Swee Hock School of Public Health, National University of Singapore and National University Health System, Singapore

^7^ Department of Surgery, Yong Loo Lin School of Medicine, National University of Singapore, Singapore

^8^ Immunology Translational Research Programme, Department of Microbiology and Immunology, Yong Loo Lin School of Medicine, National University of Singapore, Singapore

^9^ Immunology Programme, Life Sciences Institute, National University of Singapore, Singapore

^10^ Division of Infectious Diseases, National University Hospital, Singapore

^11^ Healthy Longevity Translational Research Program, Yong Loo Lin School of Medicine, National University of Singapore, Singapore.

**Corresponding author:**

Raymond Chee Seong Seet, MBBS, FRCP

Department of Medicine, Yong Loo Lin School of Medicine, National University of Singapore, Level 10, NUHS Tower Block, 1E Kent Ridge Road. Singapore 119228

Email: [raymond_seet@nus.edu.sg](mailto:raymond_seet@nus.edu.sg)

Tel: 65-67722597, Fax: 65-68724101

**ClinicalTrials.gov number:** NCT04446104

**Key words:** SARS-CoV-2 infection, zinc, vitamin C, neutralizing antibodies, transitional B cells, double-negative T cells

**Methods**

*Trial design and participants*

Asymptomatic dormitory residents at Tuas South Dormitory, Singapore, between 21 and 60 years old, who were willing to adhere to the study protocol, and return for a review 42-days later, were invited to participate in this study. Prospective participants with respiratory symptoms (e.g. fever, cough, runny nose, sore throat and/or shortness of breath), dysgeusia or anosmia, anytime within 30 days prior to their enrolment were excluded. The outbreak in this dormitory was part of a larger outbreak involving migrant workers in Singapore linked to a large hypermarket frequented by these workers.^1^ The first case at Tuas South Dormitory was reported on 7 April 2020 and unlinked cases were subsequently detected sporadically in different blocks and floors with no discernible pattern. Recruitment into the trial began 5-6 weeks later, on 18 May 2020, and a recruitment window of 14 days was set to include as many uninfected participants as possible. Information on demography (age and gender), country of origin, and medical history were obtained by direct interview and entered using FormSG, an encrypted tool developed by Singapore’s GovTech Data Science & Artificial Intelligence Capability Centre. Body weight, height, blood pressure and heart rate were measured in all participants. The trial was approved by the Domain-Specific Review Board, National Healthcare Group (2020/00561), the Ministry of Health, the multi-ministerial Joint Task Force, and was conducted under a Clinical Trial Authorization (CTA2000053) by the Health Sciences Authority which oversees all clinical trials in Singapore.

After randomization, each participant received a 42-day supply of medications and was responsible for taking the medication as instructed (except for ivermectin that was administered as a single dose). Hydroxychloroquine was sourced as hydroxychloroquine sulfate from Shanghai Pharmaceuticals Holding Co., Ltd in 100mg tablets, ivermectin from Edenbridge Pharmaceuticals LLC as 3mg tablets, povidone-iodine as povidone-iodine 0.45% (Betadine^®^) from Mundipharma Pte Ltd, zinc was sourced as zinc oxide 40mg in combination with vitamin C 250mg from Blackmores Ltd and vitamin C in 500mg tablets also from Blackmores Ltd. The number of participants was not balanced between intervention groups as infection status could only be ascertained after their enrolment, as validated and licensed serological assays were not licensed in Singapore in May 2020 when the enrolment began. Study participants who were seropositive were offered enrolment into the present substudy while those who were seronegative were enrolled into the primary DORM trial. Eighty percent participants reported nearly full compliance which was verified by counting the number of remaining pills on day 42.

*Laboratory analyses*

The Elecsys^®^ is a binding double-antigen sandwich electrochemiluminescence immunoassay that quantifies mostly IgG to the SARS-CoV-2 spike protein receptor binding domain (RBD) and was measured using the Cobas e411 analyzer (Roche, Germany).^2^ The Elecsys^®^ assay is reported in units per ml and has a measuring range of 0.40 to 250 U/ml (up to 2,500 U/ml with on-board 1:10 dilution), where a concentration of >0.80 U/ml is used to define spike antibody positivity.^2^ The cPass^TM^ assay is a surrogate virus neutralization test (sVNT) that measures non-binding neutralizing antibodies against the SARS-CoV-2 spike RBD protein and the extracellular domain of the human angiotensin-converting enzyme 2 (hACE2).^3^ The cPass^TM^ assay is reported as percent inhibition (%) of RBD-hACE2 binding where a percent inhibition of >30% has been calibrated against a high-stringency plaque reduction neutralization test to indicate adequate SARS-CoV-2 RBD-interacting neutralizing antibodies blocking the RBD-hACE2 binding.^3^ These assays were selected as they were both clinically validated, licensed by regulatory authorities and allow for high-throughput measurements in non-BSL3 laboratories.^2,3^

The DURAClone IM Phenotyping Basic kit delineates principal lymphocyte subsets as well as subpopulations of monocytes (including CD3, CD4, CD8, CD14, CD16, CD19, CD45, CD56), whereas the DURAClone IM B Cell Panel measures late maturation stages of B cells, such as transitional stage, isotype class-switch, naïve and memory stages (CD19, CD21, CD24, CD27, CD38, CD45, IgM and IgD) (Beckman, United States). Gating strategies to define different immune cell populations were based on manufacturer’s recommendations, expressed as percent (%) (Supplementary Figs. 1A and B). For logistical reasons, immunophenotyping was performed in whole blood that reached the research laboratory before a daily cutoff of 1400hrs.

*Statistical analyses*

Categorical variables are summarized as numbers (n) and percentages (%). Continuous variables are expressed as mean (standard deviation) or median (interquartile range, IQR), and, based on their distribution, compared using the one-way ANOVA and Kruskal-Wallis tests as appropriate. Categorical variables were compared using the chi-squared test. To account for multiple testing, a Bonferroni-corrected level of α=0.0125 was used to determine statistical significance. One-sample Kolmogorov-Smirnov test was used to compare differences in antibody levels from a test-value of 0. Spearman correlation analyses were performed to compare antibody levels, serum zinc and immune cells. There was no sample size calculation as this post-hoc analysis was conducted on individuals excluded from the primary prevention study in the main DORM trial.

*Study limitations*

First, we ascertained serological status of the study participants after the study was completed once commercial assays were licensed in Singapore. As such, we were unable to diagnose seropositivity in real-time after recruitment and could not balance the number of participants across different interventions. Second, we did not employ gold-standard methods of direct virus neutralization tests to estimate neutralizing antibodies. Instead, we relied on a validated surrogate virus neutralization assay that allowed for high-throughput analysis in a non-BSL3 laboratory setting. Third, we were unable to ascertain the exact timing of infection in this cohort who were predominantly asymptomatic. Although the first case identified in the dormitory was approximately 5-6 weeks before the study, it is likely that seropositive individuals were infected at different times and were at different trajectories of antibody production before their enrolment. Fourth, as we recruited only young and healthy men, results may vary when the different interventions are studied in older age groups, women and those with comorbidities. Fifth, we were unable to exclude the possibility that room contacts of study participants may themselves harbor asymptomatic illness and shed the virus during the study, providing natural boosters capable of priming immune cells to produce more antibodies.

**References:**

1. Tan THY, Toh M, Vasoo S, et al. Coronavirus Disease 2019 (COVID-19): The Singapore Experience. A Review of the First Eight Months. *Ann Acad Med Singap.* 2020;49(10):764-778.

2. Higgins V, Fabros A, Kulasingam V. Quantitative Measurement of Anti-SARS-CoV-2 Antibodies: Analytical and Clinical Evaluation. *J Clin Microbiol.* 2021;59(4).

3. Taylor SC, Hurst B, Charlton CL, et al. A New SARS-CoV-2 Dual-Purpose Serology Test: Highly Accurate Infection Tracing and Neutralizing Antibody Response Detection. *J Clin Microbiol.* 2021;59(4).
