# Supplementary material for: Zinc and vitamin C intake increases spike and neutralising antibody production following SARS‐CoV‐2 infection
Source: Clin Transl Med. 2022 Feb 20;12(2):e731. doi: 10.1002/ctm2.731 (PMC8858613; doi:10.1002/ctm2.731)

Supplementary Figure 1. Screening and randomization. From the 4,257 men who were assessed for eligibility, 3,835 were excluded: 3,789 were seronegative on recruitment, 22 withdrew from study, and 24 did not return for follow-up visit. A total of 422 men formed the primary cohort (68 received oral zinc+vitamin C, 67 oral hydroxychloroquine, 99 oral ivermectin, 107 povidone-iodine throat spray, and 81 oral vitamin C).

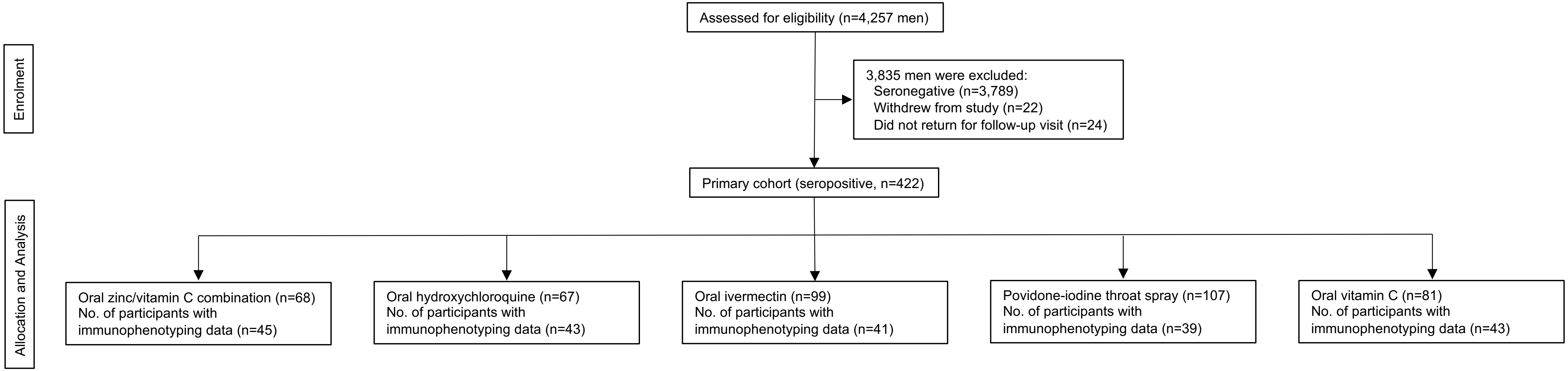

Supplementary Figure 2.

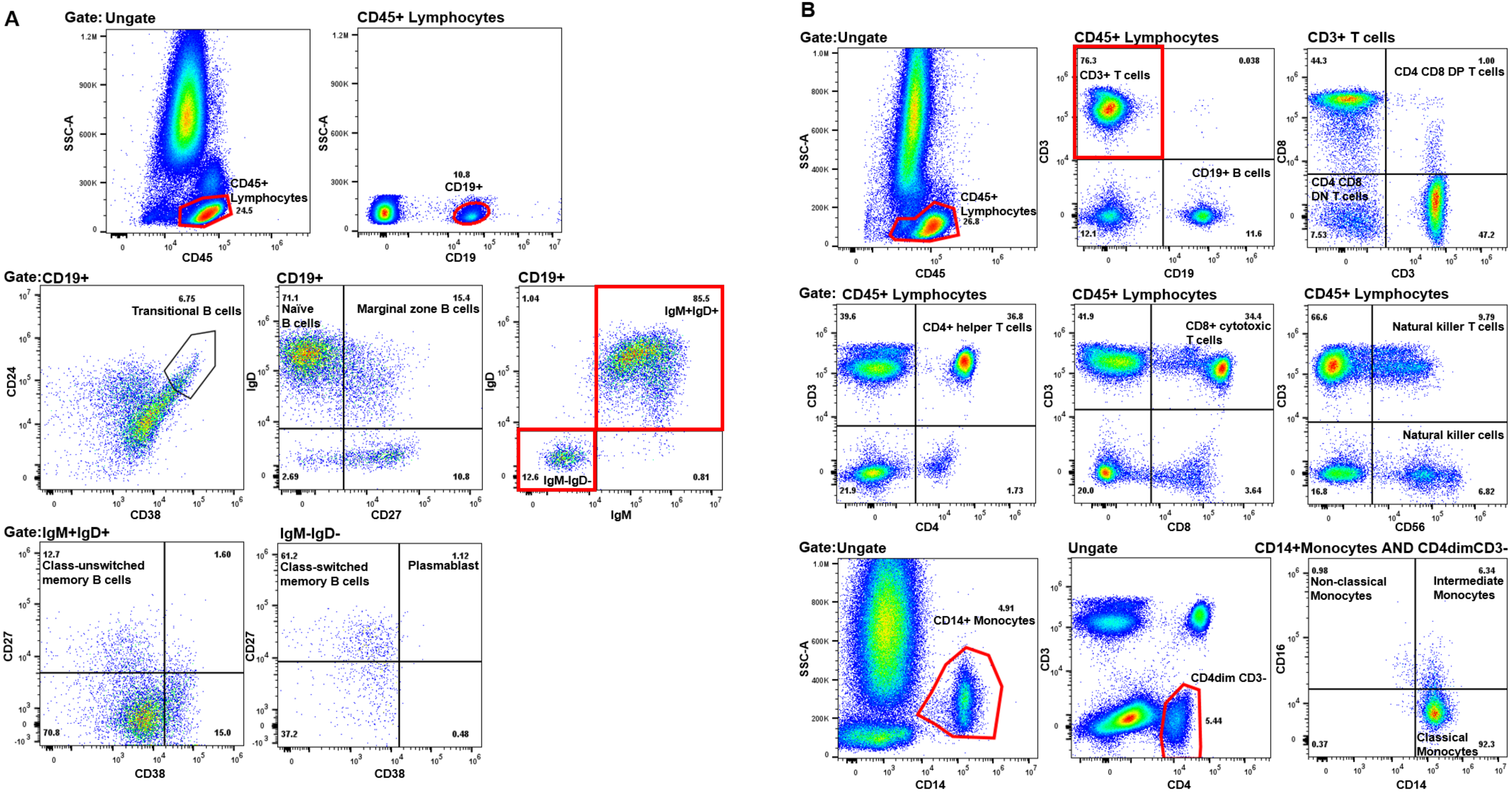

Supplementary Figure 3

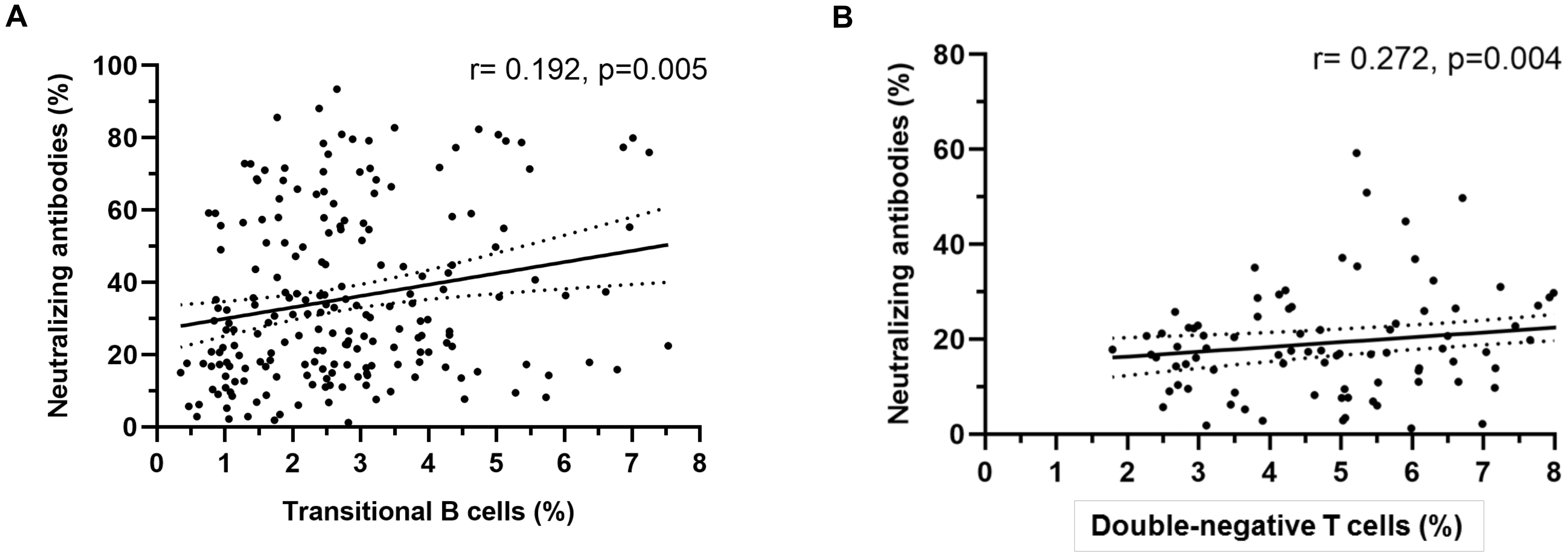

Supplement: Supplementary file 2 — Figure S1. Screening and randomisation. From the 4257 men who were assessed for eligibility, 3835 were excluded: 3789 were seronegative on recruitment, 22 withdrew from study and 24 did not return for follow‐up visit. A total of 422 men formed the primary cohort (68 received oral zinc + vitamin C, 67 oral hydroxychloroquine, 99 oral ivermectin, 107 povidone‐iodine throat spray and 81 oral vitamin C) Figure S2. Characterisation of B and T cell populations by flow cytometric methods to derive (A) transitional, class unswitched and switched, marginal zone, naïve B cells and plasmablasts, and (B) CD4+ helper, CD8+ cytotoxic, double‐negative, double‐positive, natural killer T cells, natural killer cells, classical, intermediate and non‐classical monocytes. Double‐negative and double‐positive T cells refer to surface expression of CD4 and CD8 Figure S3. (A) Scatter plot comparing the association between transitional B cells and neutralising antibodies in a subset of individuals with immunophenotyping data (n = 211). (B) Scatter plot comparing the association between double‐negative T cells and neutralising antibodies by day 42 among participants without neutralising antibodies at baseline. Neutralising antibodies were measured as percent inhibition using a surrogate virus neutralisation test (%). Solid line on each figure indicates a linear regression line and its 95% confidence interval (dotted line). Spearman correlation methods were used to derive correlation factor (r) and p‐values [file CTM2-12-e731-s002.pdf]
