# Supplementary material for: Zinc and vitamin C intake increases spike and neutralising antibody production following SARS‐CoV‐2 infection
Source: Clin Transl Med. 2022 Feb 20;12(2):e731. doi: 10.1002/ctm2.731 (PMC8858613; doi:10.1002/ctm2.731)
Supplement: Supplementary file 3 — Table S1. Comparison in immunophenotyping profile between zinc + vitamin C and other interventions on day 42. B and T cell populations were characterised in a proportion of participants following the 42‐day trial and summarised as percent (%) Table S2. Immunophenotyping and neutralising antibodies among individuals with and without seroconversion. B and T cell populations were characterised on day 42 in men who were initially negative for neutralising antibodies and summarised as percent (%). Neutralising antibodies were measured as percent inhibition using a surrogate virus neutralisation test (%) [file CTM2-12-e731-s003.docx]

**Supplementary Table 1.** Comparison in immunophenotyping profile between zinc+vitamin C and other interventions on day 42. B and T cell populations were characterized in a proportion of participants following the 42-day trial and summarized as percent (%).

|  | **Zinc+vitamin C (n=46)** | **Others**  **(n=165)** | **P-values** |
| --- | --- | --- | --- |
| CD19+ B cells (%) | 13.2 (5.1) | 12.4 (4.8) | 0.273 |
| Transitional B cells (%) | 3.49 (1.71) | 2.54 (1.39) | <0.001 |
| Class-unswitched memory B cells (%) | 8.55 (4.83) | 9.23 (5.22) | 0.426 |
| Class-switched memory B cells (%) | 71.4 (8.7) | 69.7 (9.1) | 0.241 |
| Marginal zone B cells (%) | 9.06 (3.74) | 9.31 (4.51) | 0.725 |
| Naïve B cells (%) | 71.7 (10.0) | 70.6 (10.4) | 0.508 |
| Plasmablast (%) | 0.81 (0.45) | 1.05 (0.82) | 0.064 |
| CD3+ T cells (%) | 70.0 (6.7) | 72.7 (8.0) | 0.074 |
| CD4+ helper T cells (%) | 37.8 (7.81) | 38.7 (6.5) | 0.433 |
| CD8+ cytotoxic T cells (%) | 28.1 (6.9) | 29.6 (7.1) | 0.216 |
| Double negative T cells (%) | 6.87 (4.18) | 7.15 (4.13) | 0.690 |
| Double positive T cells (%) | 1.29 (1.43) | 4.18 (4.13) | 0.332 |
| Natural killer cells (%) | 15.2 (7.6) | 13.6 (7.2) | 0.184 |
| Natural killer T cells (%) | 8.54 (8.05) | 8.15 (4.54) | 0.613 |
| Classical monocytes (%) | 87.9 (3.6) | 87.7 (4.5) | 0.731 |
| Intermediate monocytes (%) | 3.90 (1.92) | 4.73 (2.75) | 0.056 |
| Non-classical monocytes (%) | 5.19 (2.81) | 4.86 (2.95) | 0.497 |

|  | **Seroconversion (n=24)** | **No serconversion (n=97)** | **P-values** |
| --- | --- | --- | --- |
| CD19+ B cells (%) | 13.0 (5.0) | 12.3 (4.5) | 0.473 |
| Transitional B cells (%) | 2.89 (1.68) | 2.54 (1.54) | 0.331 |
| Class-unswitched memory B cells (%) | 8.90 (4.56) | 9.29 (5.02) | 0.729 |
| Class-switched memory B cells (%) | 72.2 (6.8) | 70.1 (8.9) | 0.287 |
| Marginal zone B cells (%) | 9.18 (2.70) | 9.30 (4.40) | 0.900 |
| Naïve B cells (%) | 69.7 (10.4) | 69.9 (9.9) | 0.895 |
| Plasmablast (%) | 0.84 (0.59) | 0.96 (0.63) | 0.402 |
| CD3+ T cells (%) | 71.4 (7.5) | 73.0 (7.4) | 0.355 |
| CD4+ helper T cells (%) | 37.4 (7.6) | 39.9 (7.0) | 0.125 |
| CD8+ cytotoxic T cells (%) | 29.3 (7.3) | 29.5 (7.3) | 0.858 |
| Double negative T cells (%) | 8.40 (5.13) | 5.92 (3.25) | 0.004 |
| Double positive T cells (%) | 1.82 (2.28) | 1.40 (1.76) | 0.330 |
| Natural killer cells (%) | 14.3 (6.9) | 13.4 (7.3) | 0.614 |
| Natural killer T cells (%) | 8.54 (5.72) | 7.71 (4.27) | 0.433 |
| Classical monocytes (%) | 88.9 (4.11) | 87.9 (4.1) | 0.297 |
| Intermediate monocytes (%) | 4.30 (2.04) | 4.79 (2.74) | 0.414 |
| Non-classical monocytes (%) | 4.09 (2.68) | 4.65 (2.76) | 0.370 |
| Neutralizing antibodies (%) |  |  |  |
| Baseline | 18.4 (9.6) | 14.6 (7.9) | 0.004 |
| Day 42 | 45.9 (14.9) | 12.6 (11.3) | <0.001 |

**Supplementary Table 2.** Immunophenotyping and neutralizing antibodies among individuals with and without seroconversion. B and T cell populations were characterized on day 42 in men who were initially negative for neutralizing antibodies and summarized as percent (%). Neutralizing antibodies were measured as percent inhibition using a surrogate virus neutralization test (%).
